# Chronic Low‐Level Lead Exposure Causes Auditory Impairment and Accelerates the Progression of Age‐Related Hearing Loss in C57BL/6J Mice

**DOI:** 10.1111/acel.70297

**Published:** 2025-11-10

**Authors:** Xue Bai, Li‐Hua Cheng, Zhi‐Bin Zhou, Kai‐Lang Zhou, Yan‐Peng Fu, Bing Liao, Mei‐Qun Wang, Xu‐Bo Chen, Hong‐Ping Chen, Yue‐Hui Liu, Kai Xu

**Affiliations:** ^1^ Department of Otolaryngology, Head and Neck Surgery, The Second Affiliated Hospital of Nanchang University, Jiangxi Medical College Nanchang University Nanchang China; ^2^ Department of Histology and Embryology, Jiangxi Medical College Nanchang University Nanchang Jiangxi China; ^3^ Department of Vascular Surgery, The Second Affiliated Hospital of Nanchang University, Jiangxi Medical College Nanchang University Nanchang China

**Keywords:** age‐related hearing loss, hair cell, lead, LONP1, mitochondrial dysfunction

## Abstract

Heavy metal ion exposure has become a global public health concern. Among them, lead is an extremely toxic heavy metal that poses serious health hazards to humans, particularly threatening vulnerable groups such as children and the elderly. Currently, the effects of chronic low‐dose lead exposure on the auditory system have yet to be reported. In this study, we established a chronic lead exposure mouse model and conducted comprehensive auditory function assessments. The results demonstrated that lead‐exposed mice developed high‐frequency hearing loss at early stages, which progressively worsened over time. Morphological examination of the inner ear revealed hair cell loss, reduced synaptic ribbon numbers, and disruption of gap junctions in lead‐exposed mice. Furthermore, immunofluorescence staining confirmed significantly decreased expression of the mitochondrial protease LONP1, along with markedly increased expression of its substrate HMGCS2, in the stria vascularis, sensory epithelium, and spiral ganglia of lead‐exposed mice. These findings indicate that chronic low‐level lead exposure causes inner ear damage and irreversible auditory dysfunction, while accelerating age‐related hearing loss in C57BL/6J mice. These preclinical results suggest that chronic lead exposure may represent a significant risk factor for age‐related hearing loss, deepen our understanding of lead‐induced auditory system impairment, and hold profound implications for preventing hearing damage in populations at high risk of lead exposure.

## Graphical abstract

Chronic low‐concentration lead exposure accelerates the development of ARHL. Lead exposure mediates damage to cochlear sensory cells via the mitochondrial protease LONP1, leading to irreversible hearing loss.

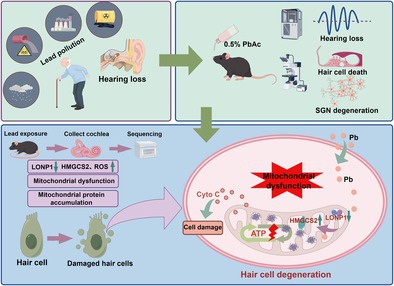

## 1 Introduction

Heavy metal ion exposure has emerged as a global public health concern, characterized by multisystemic health hazards with cumulative and delayed effects. These threats are particularly acute for vulnerable populations, including children and the elderly (Rehman et al. 2018). Major sources of pollution encompass mineral mining, industrial emissions, agricultural fertilizers, and products containing heavy metals, among which lead (Pb) stands out as one of the most prevalent and toxic metals in the environment (Klotz and Göen 2017; Gidlow 2015). Lead is released into air, soil, and drinking water through industrial discharges, lead‐based pigments, and household items like chemical batteries (Zeng et al. 2020; Bindler 2011). Owing to the bioaccumulative capacity of toxic metals, prolonged exposure to even low concentrations of lead can cause chronic systemic damage. Consequently, lead toxicity has garnered significant research interest due to its diverse exposure routes and complex mechanistic impacts (Levin et al. 2021).

Heavy metal lead is a potent systemic toxicant capable of inducing damage to multiple organ systems, including the nervous, cardiovascular, hematopoietic, and reproductive systems (Yao et al. 2021). After ingestion, lead distributes to various organs via the bloodstream. The half‐life of lead is approximately 30 days in the blood, but extends to 2 years in the brain and can persist for decades in the bones (Lidsky and Schneider 2003). The nervous system is particularly vulnerable to lead toxicity due to the metal's ability to cross the blood–brain barrier (Zahoor et al. 2024). Evidence indicates that blood lead concentrations ≥ 5 μg/dL in children can lead to cognitive decline, attention deficit hyperactivity disorder, and impaired learning capacity (Sindhu and Sutherling 2015; Guo et al. 2019). Additionally, lead exposure has been linked to an increased risk of neurodegenerative disorders, such as Alzheimer's disease and Parkinson's disease (Araki et al. 2000; Huang et al. 2024). Chronic lead exposure is also recognized as a risk factor for cardiovascular diseases, including hypertension, arrhythmias, and atherosclerosis (Pan, Gong, and Liang 2024; Shvachiy, Amaro‐Leal, et al. 2023). Epidemiological studies have shown that exposure to high concentrations of lead can cause severe hearing loss, which is often accompanied by systemic complications such as neurological and renal damage. These effects typically prompt medical attention only when multiple systems are compromised. Research by Curhan et al. has demonstrated that lead exposure increases the risk of high‐frequency hearing loss in adolescents within the US population (Shargorodsky et al. 2011). Clinical studies by Liu et al. further indicate that exposure to lead and cadmium from e‐waste sources contributes to hearing impairment in children (Liu et al. 2018). A series of animal studies have further corroborated that high‐concentration lead exposure induces acute hearing loss in rats and guinea pigs, accompanied by cochlear hair cell damage (Liu et al. 2013, 2011). Mechanistic research by Klimpel et al. revealed that lead exposure impairs cochlear auditory nerve conduction through oxidative damage and apoptosis in sensory cells, ultimately leading to severe hearing loss (Klimpel et al. 2017). However, the effects of chronic low‐level lead exposure on the auditory system remain unreported. In daily life, individuals are often exposed to low concentrations of lead from industrial pollution or dietary intake, typically resulting in subclinical lead poisoning that is difficult to detect. Therefore, elucidating the impact of chronic low‐level lead exposure on the auditory system and its underlying molecular mechanisms represents a critical gap in current research.

Age‐related hearing loss (ARHL), also known as presbycusis, is a sensorineural hearing impairment caused by degenerative changes in the auditory system due to aging. It has become a critical public health issue in aging societies worldwide. According to the World Health Organization, approximately one‐third of people aged 65 and older are affected by hearing loss globally, with the prevalence rate reaching 80% among those aged 80 and above (Rodríguez‐Valiente et al. 2020). The development of ARHL is influenced by a combination of exogenous factors, such as noise exposure and ototoxic medication, and endogenous factors (Pan, Li, et al. 2024). Among exogenous risks, exposure to heavy metal ions, especially lead, has been recognized as a potential contributor. Notably, lead exhibits cellular toxicity that shares similar pathological mechanisms with presbycusis. Consequently, chronic lead exposure may elevate the risk of hearing impairment in older adults or accelerate the progression of ARHL. In this study, we analyzed the correlation between hearing ability and blood lead concentrations in elderly individuals from the National Health and Nutrition Examination Survey (NHANES) database. The results revealed a positive correlation between blood lead levels and hearing decline. To causally investigate this association, a chronic lead exposure mouse model was established by administering lead acetate (PbAc) containing deionized water. Dynamic assessments of auditory function across different age groups demonstrated that lead exposed mice developed high frequency hearing loss at an earlier stage, with progressive worsening over time, suggesting that lead exposure accelerates the onset of ARHL. Morphological and molecular analyses further uncovered that lead exposure disrupted the mitochondrial structure of stria vascularis, hair cells, and spiral ganglion cells. This was accompanied by a significant downregulation of the mitochondrial protease LONP1 and a concomitant increase in its substrate HMGCS2 within the cochlea. In conclusion, our study demonstrates that chronic low‐level lead exposure accelerates ARHL progression, likely through impairing mitochondrial dynamics and protein homeostasis. These findings provide novel insights into the mechanisms of lead‐induced hearing loss and underscore the importance of mitigating lead exposure for hearing preservation in vulnerable populations.

## 2 Materials and Methods

### 2.1 Mouse Model

All animal experiments utilized 8‐week‐old male C57BL/6J mice purchased from the Animal Research Center of Nanchang University. Newborn mice were generated through timed mating of adult breeders. All animals were housed in a specific pathogen‐free facility with constant temperature and humidity, and provided ad libitum access to food and water. PbAc administration via drinking water is a commonly used method for establishing lead exposure models. Control group mice received standard feeding, while the lead‐exposed group was administered deionized water containing 0.5% PbAc. All experimental procedures complied with the National Institutes of Health (NIH) Guidelines for the Care and Use of Laboratory Animals and were approved by the Ethics Committee of Nanchang University. Every effort was made to minimize both the number of animals used and their discomfort throughout the study.

### 2.2 Hearing Evaluation

Auditory brainstem response (ABR) and distortion product otoacoustic emission (DPOAE) testing were performed to evaluate auditory function in mice (Xu, Chen, et al. 2022). Prior to testing, mice were deeply anesthetized, with active and reference electrodes inserted near the calvarium and pinna, respectively. All procedures were conducted in a soundproof chamber, with body temperature maintained using a heating pad. For ABR measurements, click stimuli at frequencies of 8, 16, 24, 32, and 40 kHz were delivered using a Tucker‐Davis Technologies system. The sound intensity began at 90 dB SPL and decreased in 10 dB steps until the waveform became undetectable, with the threshold defined as the lowest intensity eliciting a reproducible response (Xu, Chen, et al. 2022). For DPOAE measurements, test frequencies were represented as the geometric mean of *f*1 and *f*2, with a ratio of *f*1:*f*2 = 1:1.2. The distortion products (2*f*1 − *f*2) were averaged over 200 recordings, and the output amplitudes of DPOAEs were measured across specified frequencies.

### 2.3 Cell Culture and Treatment

The cell culture experiments were conducted in accordance with our previously published protocols (Zhou et al. 2025). Briefly, HEI‐OC1 cells were cultured in a high‐glucose DMEM containing 10% fetal bovine serum (Gibco, 11054001; Thermo Fisher Scientific). Cells were maintained in a 5% CO_2_ atmosphere at 37°C in an incubator for 24 h, followed by microscopic examination to assess density and viability. For treatment, the culture medium was replaced with fresh medium containing PbAc at concentrations of 5 μg/mL or 10 μg/mL for 24 h. After removing the PbAc‐containing medium, the cell viability of cells in different treatment groups was evaluated using the Cell Counting Kit‐8 (CCK‐8), and the experimental steps were strictly carried out in accordance with the instructions.

### 2.4 Cochlear Culture and Treatment

Cochleae were rapidly dissected from euthanized postnatal Day 3 C57BL/6J mice in ice‐cold Hank's balanced salt solution. Under a stereomicroscope, the basilar membranes were carefully isolated. The dissected basilar membranes were maintained in culture medium containing 10% bovine serum albumin, 2 mM glutamine, 1× Insulin‐Transferrin‐Selenium supplement, and 50 μg/mL ampicillin at 37°C. Tissue integrity and viability were assessed daily using phase‐contrast microscopy. To establish the lead injury model, the basilar membranes were treated with PbAc at concentrations of 5 or 10 μg/mL for 24 h, followed by collection for subsequent analyses.

### 2.5 Immunofluorescence

Cochlear explants or HEI‐OC‐1 cells were fixed with 4% paraformaldehyde in phosphate‐buffered saline (PBS) for 20–30 min at room temperature, followed by three PBS washes to remove residual fixative. Samples were permeabilized with 0.3% Triton X‐100 and blocked with PBS containing 5% bovine serum albumin (BSA) for 1 h to minimize nonspecific binding, then incubated overnight with a mouse anti‐Cx26 (1:200 dilution, 33‐5800, Invitrogen), mouse anti‐CtBP2 (1:200, 612044, BD Biosciences), polyclonal rabbit anti‐myosin7a (1:500 dilution, 256790, Proteus Bio‐Sciences), rabbit anti‐LONP1 (1:200, HPA002192, Sigma‐Aldrich), rabbit anti‐HMGCS2 (1:200, A14244, Abclonal), mouse anti‐beta III Tubulin (1:500, ab78078, abcam). After three PBS washes, samples were incubated with fluorophore‐conjugated secondary antibodies for 1 h at room temperature in the dark. Nuclei were counterstained with DAPI (Sigma‐Aldrich, D9542) for 10 min. The samples were treated with anti‐quenching fluorescent mounting agents. Images were acquired using a confocal microscope (Olympus FV1000; Olympus, Tokyo, Japan) with standardized exposure settings across experimental groups.

### 2.6 Transcriptome Analysis

RNA sequencing (RNA‐Seq) was performed to compare the transcriptomes of the control group and the lead exposed group. First, cochlear tissues were isolated, and total RNA was extracted from the cochlea for RNA‐Seq analysis. Subsequently, library preparation was carried out, including nucleic acid fragmentation, adapter ligation, and amplification. Sequencing analysis was conducted using the Illumina NovaSeq6000 platform. Differential expression analysis and pathway enrichment analysis were performed on the sequencing data. All top‐ranked gene sets were manually curated to confirm their accurate functional and pathway classifications.

### 2.7 RNA Extraction and Real‐Time Quantitative PCR

Cochlear tissues from mice were physically homogenized in TRIzol Reagent (Thermo Fisher Scientific, 15596026). The lysate was incubated at room temperature for 10 min. Chloroform was added, mixed thoroughly, and centrifuged for 15 min. The aqueous phase was collected, combined with 0.5 mL isopropanol, and incubated for 10 min. After centrifugation, the RNA pellet was washed twice with 75% ethanol, air‐dried, and dissolved in RNase‐free water to obtain total cochlear RNA. Reverse transcription was performed using the PrimeScript RT Reagent Kit (Takara Bio Inc.). Specific primers and SYBR Premix Ex Taq (Takara, Japan) were utilized for quantitative PCR on a StepOnePlus Real‐Time PCR System (Applied Biosystems, Foster City, CA, USA). Relative gene expression was calculated using the 2^−ΔΔCt^ method, normalized to the housekeeping gene GAPDH.

### 2.8 Western Blotting

Cochlear tissues or cells were homogenized by sonication in ice‐cold RIPA lysis buffer supplemented with protease inhibitor cocktail and phenylmethylsulfonyl fluoride, followed by incubation on ice for 15 min. After centrifugation at 4°C for 5 min, the supernatant containing protein extract was collected. Equal amounts of protein samples were mixed with 5× Laemmli loading buffer, separated by electrophoresis, and transferred to a polyvinylidene fluoride (PVDF) membrane. The membrane was incubated overnight at 4°C with specific primary antibodies, followed by three 10‐min TBST washes. A horseradish peroxidase‐conjugated secondary antibody was then applied for 1.5 h at room temperature. Protein bands were visualized using enhanced chemiluminescence substrate and imaged with a ChemiDoc XRS system. Semi‐quantitative analysis was performed using ImageJ software (NIH, Bethesda, MD, USA).

### 2.9 Transmission Electron Microscope

Cochlear tissue samples were fixed in 2.5% glutaraldehyde (Sigma‐Aldrich, G5882) in 0.1 M phosphate buffer (pH 7.4) at 4°C for 4 h. After fixation, decalcification was performed in 10% disodium EDTA for 48 h, followed by post‐fixation with 1% osmium tetroxide (Sigma‐Aldrich, 05500) for 2 h. The fixed tissues were dehydrated through a graded ethanol series and rinsed twice in acetone for 5 min each. Samples were embedded in fresh epoxy resin, sectioned into ultrathin slices, and stained with uranyl acetate and Reynolds' lead citrate (Sigma‐Aldrich, 15326). Images were acquired using a transmission electron microscope (FEI Tecnai G220 TWIN, USA).

### 2.10 Statistical Analysis

Statistical analysis was performed using GraphPad Prism 8.0 (GraphPad Software, USA). All data are expressed as mean ± standard deviation (SD). Prior to analysis, normality and homogeneity of variance were assessed across all groups. For comparisons between two independent groups, a two‐tailed unpaired Student's *t*‐test was performed to assess statistical significance. For comparisons involving more than two groups, one‐way analysis of variance (ANOVA) was applied under the assumptions of normal distribution and homogeneity of variance, and a *p* < 0.05 indicated statistical significance.

## 3 Results

### 3.1 Hearing Loss in the Elderly Population Is Associated With Serum Lead Levels

**FIGURE 1:**
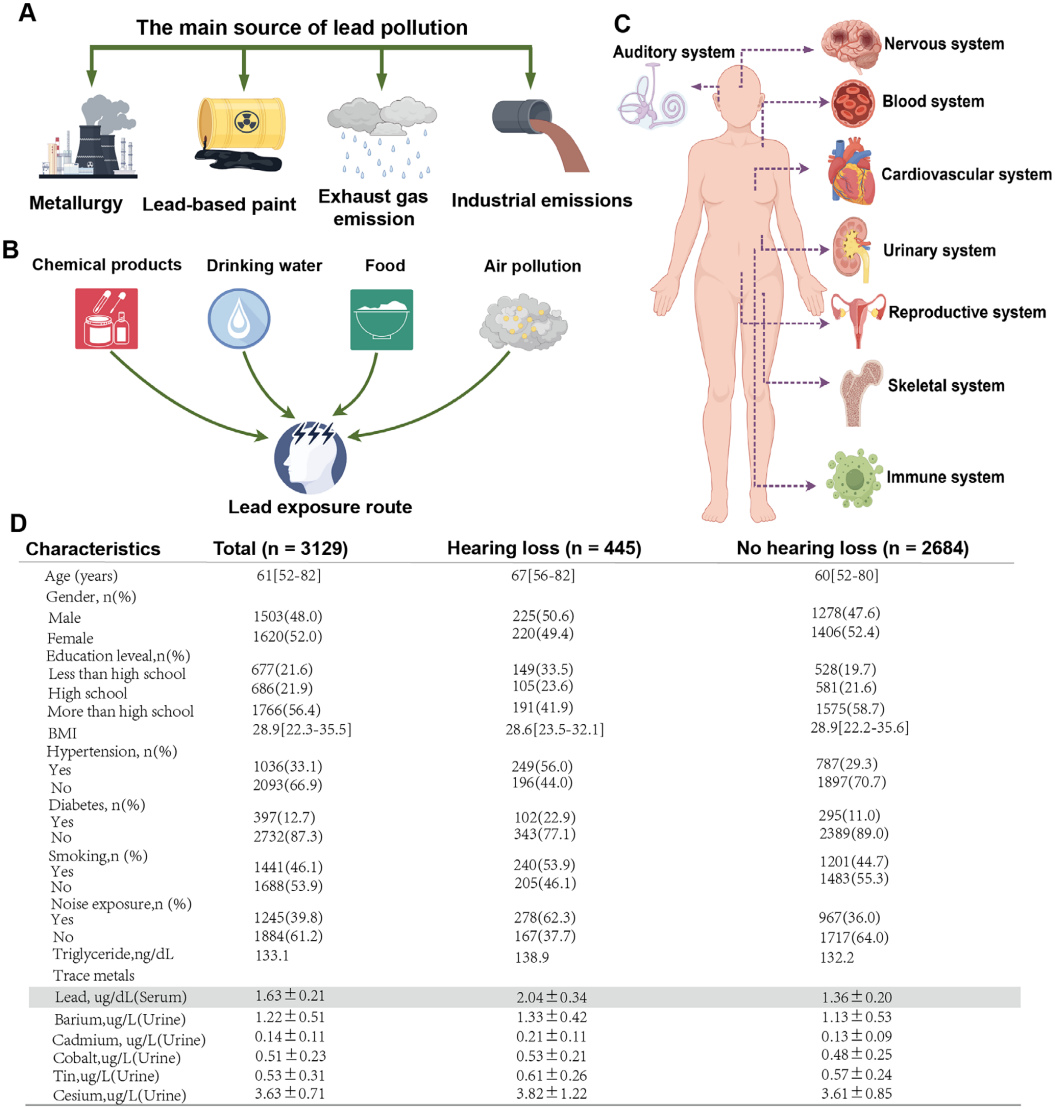
The serum lead content in the population with hearing loss is significantly increased. (A) The main sources of lead pollution. (B) The main ways for the population to be exposed to lead. (C) The main hazards of lead exposure to various systems of the human body. (D) The baseline characteristics of the study population in the database, among which the serum lead concentration of the population with hearing loss was significantly increased.

Trace metal elements play critical roles in numerous biochemical processes and are essential for the synthesis of enzymes that regulate cellular homeostasis and energy production (Cannas et al. 2020). However, excessive exposure to trace metals can induce a spectrum of adverse health effects, with lead (Pb) being one of the most abundant and cytotoxic metals in the environment. Environmental lead contamination primarily originates from anthropogenic activities such as mining operations, waste disposal, industrial effluents, and vehicular emissions (Figure 1A) (Ardila et al. 2023; Rahman and Singh 2019). Due to its widespread use, humans are exposed to exogenous lead through ingestion, inhalation (e.g., contaminated water, food, air), or direct contact with lead‐containing chemicals (Figure 1B). Studies have demonstrated that chronic or acute lead exposure causes multi‐system damage, including neurological, reproductive, cardiovascular, immune, and hematopoietic dysfunction (Figure 1C). High‐concentration lead exposure is strongly associated with severe hearing loss, particularly in children, whose auditory systems are highly vulnerable (Solis‐Angeles et al. 2021). However, the relationship between low‐level chronic lead exposure and hearing impairment remains unclear. To address this, we analyzed comprehensive data from 3129 participants in the NHANES database, focusing on the association between serum trace metal concentrations and hearing loss in older adults. Hearing loss was defined as an average pure‐tone hearing threshold increase of ≥ 25 dB (Olusanya et al. 2019). Notably, serum lead concentrations were significantly higher in the hearing loss group compared to individuals without hearing impairment (Figure 1D). These findings suggest that hearing loss in the elderly may be associated with chronic low‐concentration lead exposure. However, large‐scale cohort studies or animal experiments demonstrating a direct causal relationship between blood lead levels and ARHL are still lacking. Therefore, this study was designed to investigate this significant scientific question.

### 3.2 Chronic Lead Exposure Accelerates ARHL in C57BL/6J Mice

**FIGURE 2:**
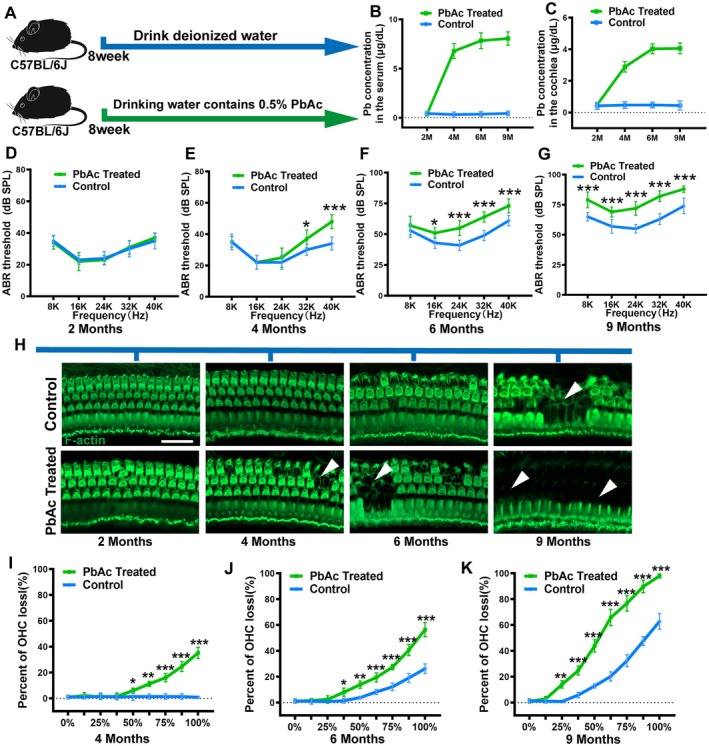
Chronic lead exposure leads to progressive hearing loss in C57BL/6J mice. (A) Construction of mouse models exposed to lead. (B) The lead content in the serum of mice with chronic lead exposure increased significantly. (C) The lead content in the cochlea of mice with chronic lead exposure increased. (D–G) Comparison of hearing thresholds between mice with chronic lead exposure and the control group. (H) Representative image of the survival of cochlear hair cells in different treatment groups. (I–K) Quantitative analysis of the loss of cochlear hair cells in mice with chronic lead exposure. *p < 0.05, **p < 0.01, ***p < 0.001. Scale in panel H represents 40 μm.

The C57BL/6J mouse strain exhibits early‐onset hearing loss due to a specific mutation in the cadherin 23 gene (Kane et al. 2012). In C57BL/6J mice, hearing impairment typically begins in the high‐frequency range at approximately 4 months of age and progressively extends to lower frequencies with aging. This auditory decline is accompanied by degenerative pathological changes in hair cells, the stria vascularis, and spiral ganglion neurons' phenotypes that closely resemble the characteristics of human ARHL. Consequently, C57BL/6J mice have long been utilized as a well‐established model in studies investigating ARHL (Sun et al. 2025, 2023; Xia et al. 2025; Xu et al. 2023). To investigate the impact of chronic low‐concentration lead exposure on ARHL, we used C57BL/6J mice and constructed a chronic lead exposure mouse model by providing them with deionized water containing 0.5% PbAc (Figure 2A). Lead levels in mouse serum and cochlea were detected using atomic absorption spectrometry. The results showed significantly elevated lead concentrations in the blood of the lead‐exposed group (Figure 2B). Similarly, lead accumulated in the cochlea of lead‐exposed mice, reaching a peak concentration of 4.08 ± 1.44 μg/dL (Figure 2C). Indeed, lead accumulation varies across different organs following exposure, which may explain its differential effects on organ function. The observed disparity in lead levels between the cochlea and serum in lead‐exposed mice may be attributed to the cochlea's complex structure as a delicate peripheral sensory organ, composed of bony tissue, multiple cell types, and fluid compartments, coupled with its relatively limited blood supply. Furthermore, the blood‐labyrinth barrier in the cochlea restricts the passage of substances from systemic circulation into the endolymph. Dynamic assessment of hearing function across treatment groups revealed that lead‐exposed mice developed hearing loss earlier. Hearing loss at high frequencies (32, 40 kHz) was observed at 4 months of age and progressively worsened over time (Figure 2D,E). At 6 and 9 months of age, the ABR thresholds of lead‐exposed mice were significantly elevated compared to the control group (Figure 2F,G). Furthermore, we assessed cochlear sensory cell survival by performing immunofluorescence staining on the basilar membrane. Consistent with the ABR results, lead‐exposed mice exhibited hair cell loss in the basal turn starting at 4 months of age (Figure 2H). This hair cell loss progressively worsened over time and spread toward the middle and apical turns. Quantitative analysis of hair cells along the entire length of the basilar membrane confirmed that the rate of hair cell loss was significantly higher in lead‐exposed mice compared to the control group (Figure 2I–K). These results indicate that chronic low‐concentration lead exposure leads to lead accumulation in the cochlea and accelerates the onset of ARHL.

### 3.3 Lead Exposure Causes Disruption of Cochlea Synapses and Gap Junctions in Mice

**FIGURE 3:**
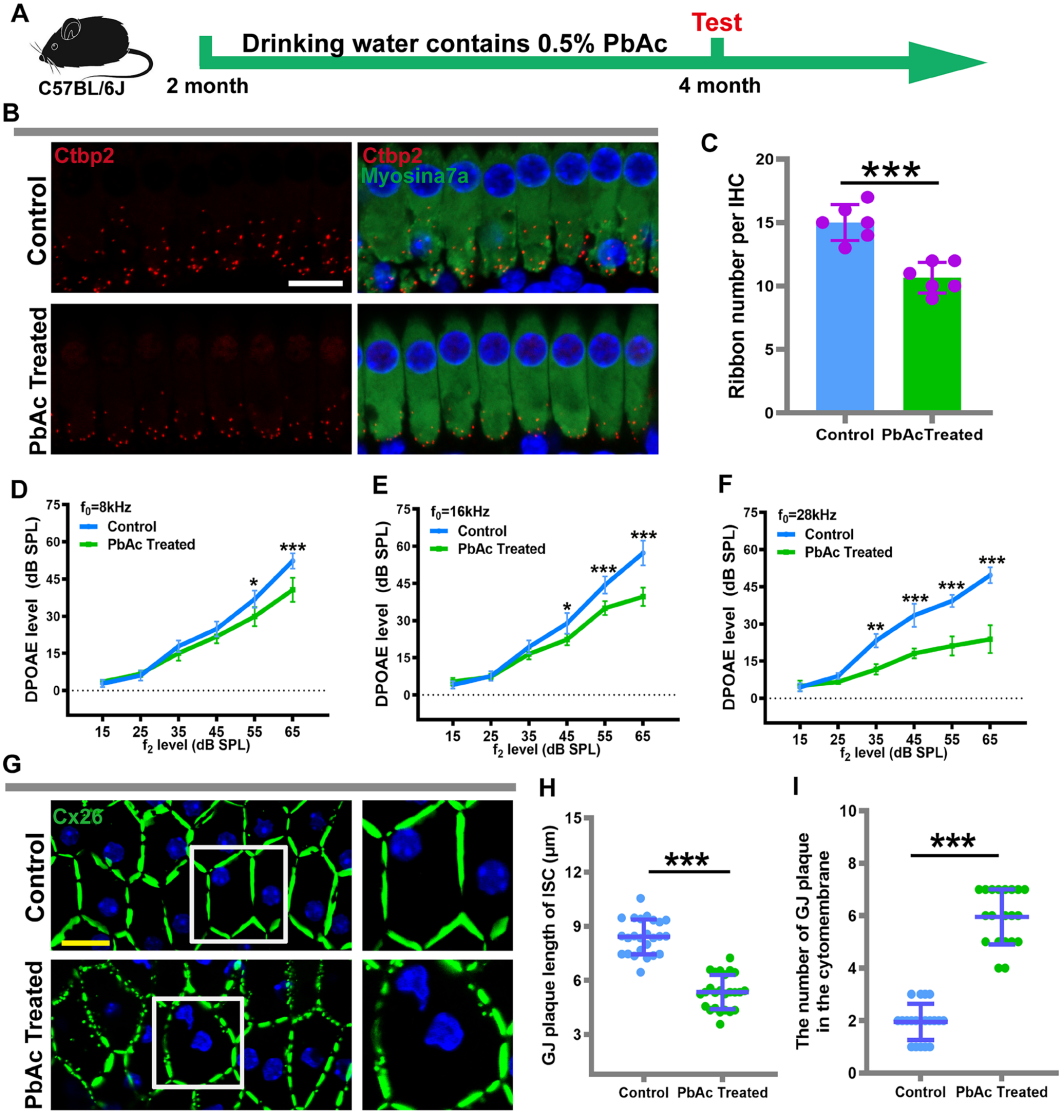
Lead exposure causes disruption of cochlea synapses and gap junctions in C57BL/6J mice. (A) Construction of mouse models exposed to lead. (B) Representative images of ribbon synapses in the inner hair cell of the control and lead exposure group. (C) Quantitative analysis of the number of ribbon synapses. (D–F) DPOAE levels were measured at different frequencies. (G) Immunofluorescence staining was used to detect the integrity of the cochlear Cx26 plaque. (H–I) Quantification of the length of GJP, and the number of Cx26 plaques along a single cell border of ISCs. *p < 0.05, **p < 0.01, ***p < 0.001. Scale in panels B and G represents 10 μm.

The core pathological changes in ARHL include not only the degeneration of hair cells but also progressive damage and loss of synapses between inner hair cells and auditory nerve fibers (Xie et al. 2024). This synaptopathy is considered one of the early features of ARHL, potentially occurring even before apparent hair cell loss and elevated hearing thresholds, also known as hidden hearing loss (Wu et al. 2019). Previous studies have reported that low‐level lead exposure can cause irreversible neural damage, posing significant harm to the developing nervous systems of children (Schneider 2023). Using immunofluorescence staining, we assessed the impact of lead exposure on hair cell synapses in mice. The results showed a significant reduction in the number of inner hair cell synapses in the middle turns of the lead exposed group (Figure 3B,C). DPOAE testing, used to evaluate the cochlear active amplification function, revealed a decline in this function in lead exposed mice, with a more pronounced decline at higher frequencies (Figure 3D–F). Gap junction channels composed of connexin 26 (Cx26) are primarily expressed in supporting cells of the cochlea, rather than directly in cochlear sensory cells (Chen et al. 2018). Studies have shown that these gap junction channels play a critical role in maintaining inner ear homeostasis and are directly involved in hearing formation by regulating potassium ion recycling (Zong et al. 2025; Wang et al. 2025). Our recent research indicates that disruption of gap junction plaques is one of the important pathological phenotypes of ARHL (Xu et al. 2023). Therefore, we investigated the effect of lead exposure on gap junction plaques in cochlear supporting cells of mice. The results demonstrated that low‐concentration lead exposure compromised the integrity of cochlear gap junction channels, causing the gap junction plaques to exhibit a fragmented appearance in inner sulcus cells of the cochlea (Figure 3G). Quantitative analysis revealed a significant decrease in the length of gap junction plaques and a significant increase in their number in the lead exposed group (Figure 3H,I). These results indicate that chronic low‐concentration lead exposure damages inner hair cell synapses and supporting cell gap junction channels, disrupting inner ear homeostasis and subsequently leading to hair cell damage and irreversible hearing loss.

### 3.4 The Potential Molecular Mechanism of Cochlear Injury Caused by Lead Exposure

**FIGURE 4:**
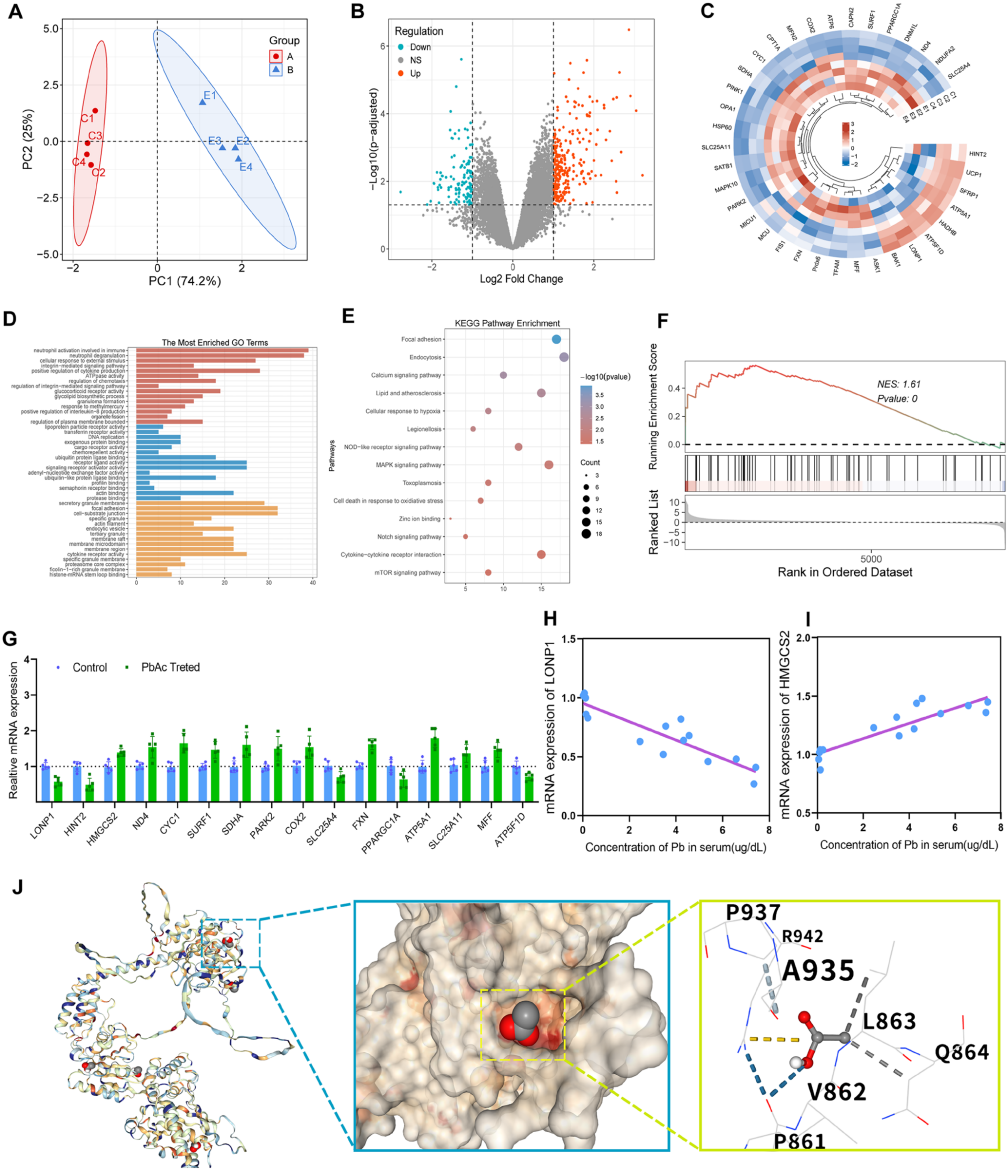
The potential molecular mechanism of cochlear injury caused by lead exposure. (A) PCA analysis of the control and lead exposure groups. (B) Volcano diagrams of differentially expressed genes. (C) The top 36 significantly altered genes in the lead exposure groups were shown. (D) GO enrichment analysis of the lead exposure groups. (E) KEGG enrichment analysis of differentially expressed genes. (F) GSE analysis of the mitochondrial protein degradation pathway. (G) Characterization of mitochondrial function‐related gene expression changes in the lead exposure groups. (H, I) Correlation analysis of lead concentration in serum and the expressions of LONP1 and HMGCS2. (J) Molecular docking simulation of lead acetate and LONP1 binding mode.

We further explored the molecular mechanisms of lead exposure‐induced cochlear tissue damage by comparing gene expression differences in the cochlea of lead‐exposed mice versus controls using transcriptomic analysis. Specifically, principal component analysis showed a total variation rate of 92.4% (Figure 4A). The volcano plot revealed 14,622 genes detected, with 143 genes significantly downregulated and 236 genes significantly upregulated (Figure 4B). The gene heatmap displayed the top 36 differentially expressed genes, showing significant alterations in the expression of a series of genes related to mitochondrial function. Among these, the expression of *LONP1*, *HINT2*, and *SLC25A4* was significantly downregulated (Figure 4C). Gene Ontology (GO) and Kyoto Encyclopedia of Genes and Genomes (KEGG) pathway enrichment analyses indicated that pathways related to mitochondrial metabolism, immune response, and cell death were enriched (Figure 4D,E). Furthermore, Gene Set Enrichment Analysis (GSEA) showed significant enrichment in the mitochondrial protein degradation pathway (Figure 4F). Real‐time quantitative PCR results validated the expression changes of the mitochondrial protease LONP1 and its substrate HMGCS2. Linear regression analysis examining the relationship between the expression of LONP1 and HMGCS2 with serum lead concentration indicated that LONP1 expression was negatively correlated with serum lead concentration, while HMGCS2 expression was positively correlated with serum lead content (Figure 4H,I). Additionally, we performed molecular docking simulations to model the binding site of Pb^2+^ with LONP1. The results clearly demonstrate that Pb^2+^ binds to the structural domain of LONP1 via hydrogen bonds. The amino acid residues adjacent to Pb^2+^ include PRO861, VAL862, LEU863, GLN864, ALA935, PRO937, and ARG942 (Figure 4J).

### 3.5 Lead Exposure Downregulates the Expression of Mitochondrial Protease LONP1 in the Cochlea

**FIGURE 5:**
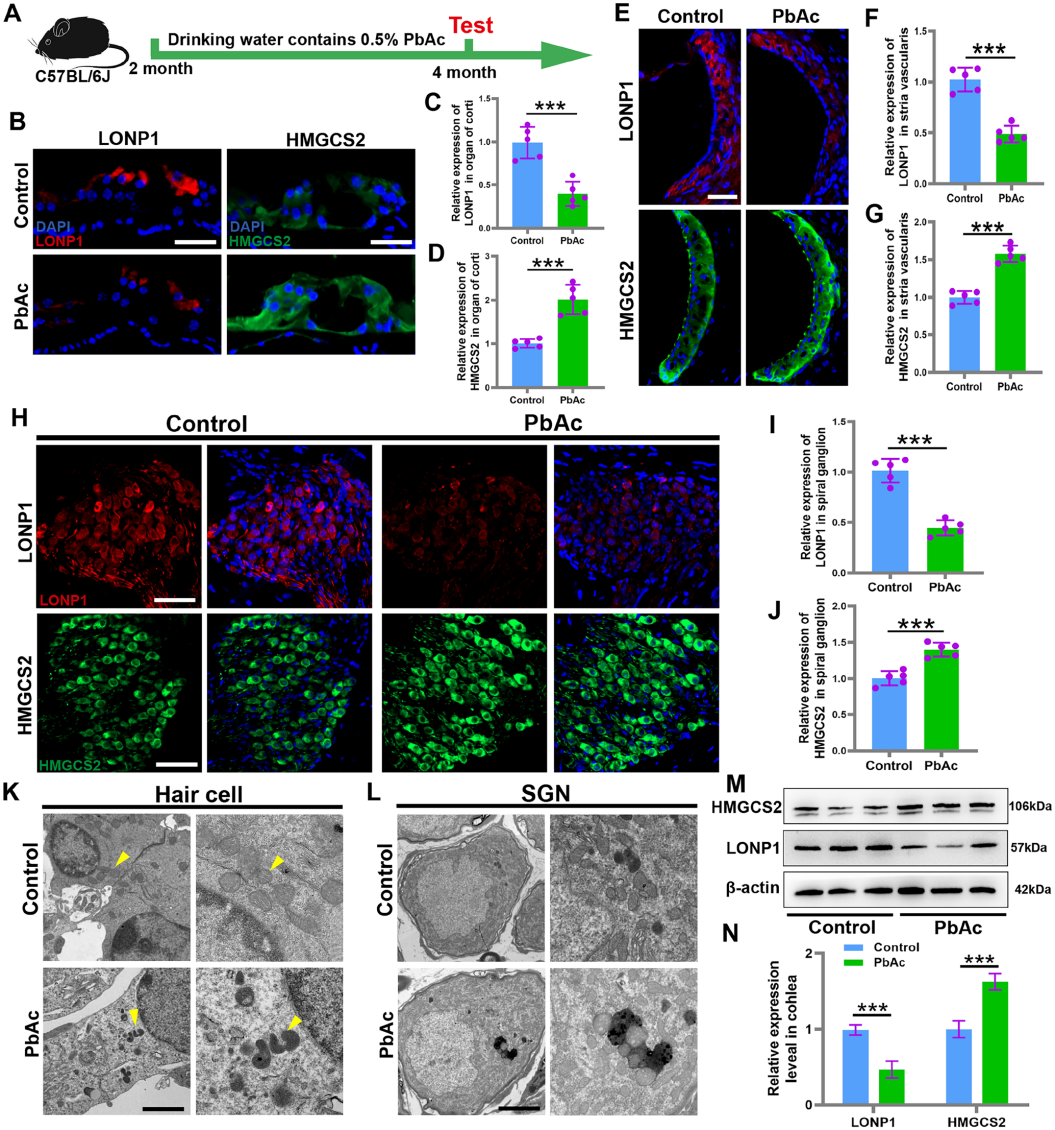
Lead exposure downregulates the expression of mitochondrial protease LONP1 in the cochlea. (A) Construction of mouse models exposed to lead. (B) Immunofluorescent staining of LONP1 (red) and HMGCS2 (green) in organ of Corti. (C, D) Quantification of immunolabeling for LONP1 and HMGCS2 in organ of Corti. (E) Immunofluorescent staining of LONP1 (red) and HMGCS2 (green) in stria vascularis. (F, G) Quantification of immunolabeling for LONP1 and HMGCS2 in stria vascularis. (H) Immunofluorescent staining of LONP1 (red) and HMGCS2 (green) in SGNs. (I, J) Quantification of immunolabeling for LONP1 and HMGCS2 in SGNs. (K, L) The ultrastructural morphology of hair cell and SGNs in the control group or lead exposure group. (M, N) Western Blot and histogram of LONP1 and HMGCS2 in cochlear. ***p < 0.001. Scale in panel B, E and H represents for 20 μm, Scale in panel K and L represents for 0.5 μm.

The mitochondrial protease LONP1 is a highly conserved ATP‐dependent protease crucial for ensuring mitochondrial protein stability and maintaining mitochondrial metabolic homeostasis (Bahat et al. 2015). Reduced expression of LONP1 leads to the accumulation of mitochondrial proteins and functional abnormalities, consequently causing cellular and tissue damage (Geisler et al. 2019). To elucidate the relationship between LONP1 and lead exposure‐induced hearing loss, we examined the expression levels of LONP1 and its substrate HMGCS2 in the cochlea of lead‐exposed mice (Figure 5A). Compared with the signal richness of the cochlea in the control group, a significant down‐regulation of LONP1 was observed in the organ of Corti of lead‐exposed mice, accompanied by an increased expression of its substrate HMGCS2 (Figure 5B–D). Furthermore, downregulated LONP1 expression and upregulated HMGCS2 expression were also observed in the stria vascularis and spiral ganglion cells (Figure 5E–J). Furthermore, we employed transmission electron microscopy to assess the impact of lead exposure on mitochondrial structure. In the hair cells of lead‐exposed mice, we observed abnormal mitochondrial morphology characterized by electron‐dense matrices and loss of normal cristae structure (Figure 5K). Within spiral ganglion neurons, lead‐exposed groups exhibited abnormal mitochondrial pigment deposits accompanied by partial mitochondrial aggregation and fusion (Figure 5L). These findings indicate that lead exposure induces mitochondrial damage in critical inner ear structures, which may represent one of the key mechanisms underlying lead‐induced auditory impairment. Additionally, semi‐quantitative Western blotting results further confirmed the decreased total protein expression of LONP1 and the increased expression of its substrate HMGCS2 in the cochlea of lead‐exposed mice (Figure 5M,N). These results indicate that lead accumulation in the cochlea may contribute to damage in key cochlear tissues by affecting LONP1 and consequently impairing the mitochondrial protein degradation system.

### 3.6 Lead Exposure Induced Degeneration of Hair Cells in Mouse Cochlear Explants

**FIGURE 6:**
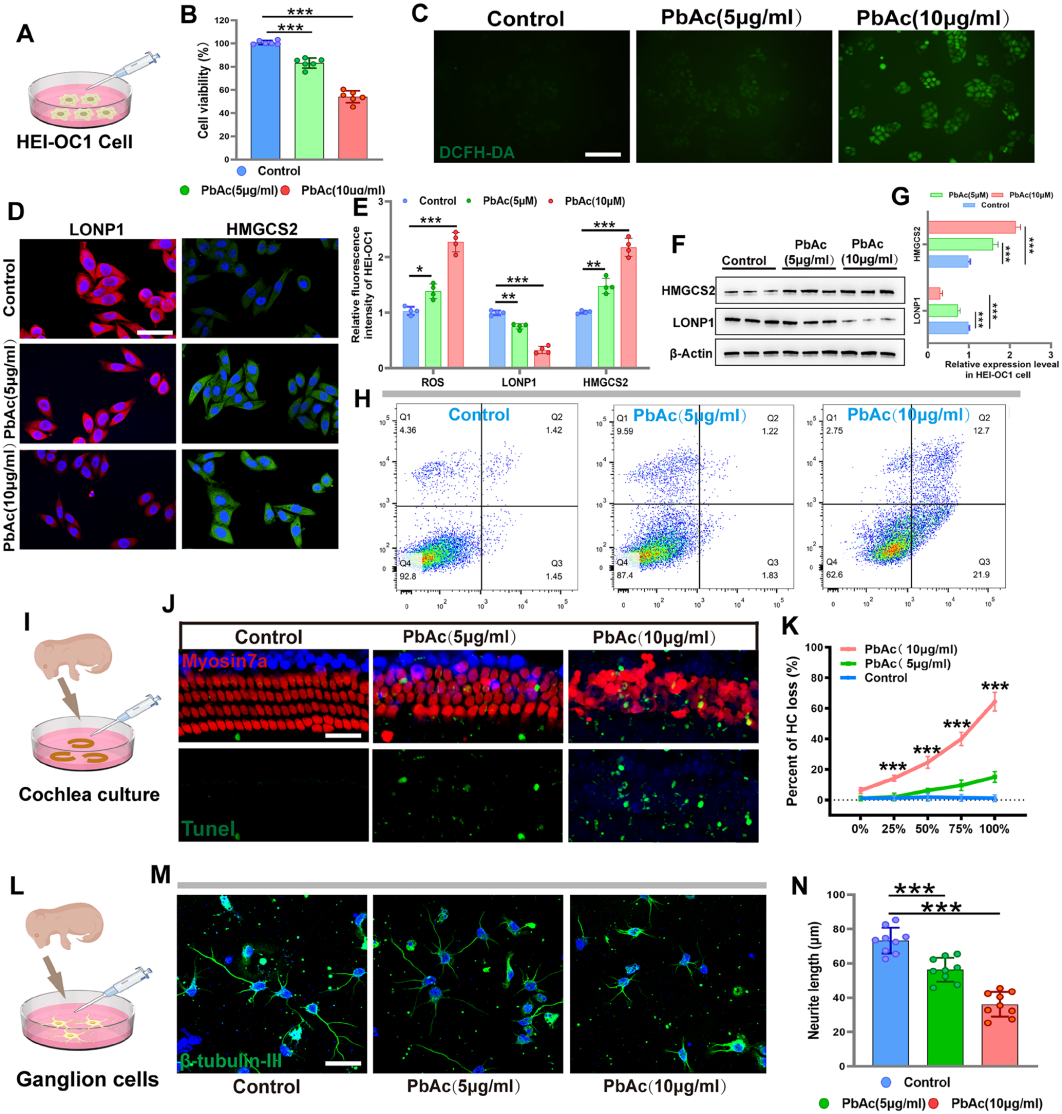
Lead exposure induced degeneration of hair cells in mouse cochlear explants. (A) Construct a lead‐exposed cell culture model. (B) Cell viability was detected by CCK8. (C) Intracellular ROS levels detected by DCFH staining. (D) The expression levels of LONP1 and HMGCS2 were detected by immunofluorescence staining. (E) Quantification of immunolabeling for LONP1 and HMGCS2 in HEI‐OC1 cells. (F, G) Western blot analysis of the expression of LONP1 and HMGCS2 in HEI‐OC1 cells. (H) Flow cytometric assessment of the apoptotic rate of OC‐1 cells after lead treatment. (I) Construct an in vitro cochlear culture lead exposure model. (J, K) Lead exposure caused destructive damage to cochlear hair cells. (L) Construct a lead exposure model for ganglion cell culture in vitro. (M, N) Lead exposure caused destructive damage to synaptic growth. *p < 0.05, **p < 0.01, ***p < 0.001. Scale in panels B, E and H represents 20 20 μm.

Construction of a lead exposure cell culture model using HEI‐OC1 cells (Figure 6A). HEI‐OC1 cells were treated with different concentrations of PbAc, and cell viability assessed by the CCK‐8 assay, showed a concentration‐dependent decrease (Figure 6B). Intracellular ROS levels were detected using the DCFH probe, and the results showed that intracellular ROS levels were significantly elevated following PbAc treatment (Figure 6C). Furthermore, immunofluorescence staining was employed to detect the expression levels of the mitochondrial protease LONP1 and its substrate HMGCS2 within the cells. The results indicated that PbAc treatment led to decreased LONP1 expression and increased HMGCS2 expression (Figure 6D,E). Western blotting results further confirmed that lead exposure affected the expression of both LONP1 and HMGCS2 (Figure 6F,G). Annexin V/PI apoptosis assays revealed a gradual increase in the proportion of apoptotic cells as the PbAc concentration increased (Figure 6H). We employed transmission electron microscopy to observe the mitochondrial morphology in HEI‐OC1 cells. Compared with the control group, the low‐concentration lead exposure group exhibited mitochondria with an electron‐dense matrix accompanied by granular deposits (Figure S1A). This phenomenon was more pronounced in the high‐concentration lead exposure group. Furthermore, JC‐1 fluorescent probe staining demonstrated a disruption of mitochondrial membrane potential in the lead‐exposed cells (Figure S1B,C). These results collectively confirm that lead exposure impairs mitochondrial function. After PbAc exposure, cochlear hair cells were labeled using Myosin7a immunofluorescence staining, and apoptotic cells were identified using TUNEL staining (Figure 6I). As shown in Figure 6J, lead exposure caused destructive damage to cochlear hair cells, with a significant number of apoptotic cells observed (Figure 6J,K). Additionally, spiral ganglion neurons were cultured (Figure 6L). Following PbAc exposure, the synaptic morphology of the ganglion neurons was examined, revealing that lead exposure impaired synaptic growth (Figure 6M). Quantitative analysis demonstrated a significant reduction in synaptic length in the PbAc‐treated ganglion neurons (Figure 6N). These results indicate that lead exposure directly damages cochlear hair cells and neural transmission, which may be a significant contributing factor to hearing loss.

## 4 Discussion

Heavy metal exposure is considered a significant risk factor for hearing loss. Long‐term or high‐concentration exposure to heavy metals can lead to severe auditory dysfunction (Yang et al. 2024). Lead, a highly toxic heavy metal, exhibits systemic toxicity and can affect multiple systems within the body, including the nervous system, cardiovascular system, hematopoietic system, and auditory and vestibular systems (Mitra et al. 2017). After ingestion, lead is distributed to various organs via the bloodstream and can accumulate in tissues, particularly in bones where its half‐life can extend for decades (Tchounwou et al. 2012). Under physiological conditions, lead primarily exists as the divalent cation Pb^2+^. The toxicity of Pb^2+^ arises from its ability to competitively substitute for other metal ions or to covalently bind to antioxidants, rendering them inactive and inducing oxidative stress (Dudev et al. 2018; Gonzalez‐Villalva et al. 2025). The US Centers for Disease Control and Prevention defines a blood lead level ≥ 5 μg/dL in adults as the occupational exposure reference value. Levels between 5 and 20 μg/dL are classified as mildly elevated, typically requiring elimination of exposure sources or nutritional intervention, while levels ≥ 20 μg/dL are defined as moderate to severe elevation, generally necessitating medical intervention. Studies have shown that high‐concentration lead poisoning causes severe hearing loss, often accompanied by complications in other systems, prompting attention and timely intervention (Roth and Salvi 2016). However, the impact of chronic, low‐level lead exposure on the auditory system remains unclear. In this study, we established a chronic lead exposure mouse model and measured serum lead levels, which were approximately 7.34 ± 1.28 μg/dL, falling within the range of mild elevation observed in human populations. This aligns with the low‐level lead exposure mouse model developed by Flores‐Montoya et al., where the average serum lead concentration was reported as 3.4 μg/dL, a value relatively close to that in our model (Dominguez et al. 2019). Furthermore, we demonstrated that chronic low‐level lead exposure induces auditory dysfunction and accelerates the progression of ARHL in C57BL/6J mice. Due to improved detection methods and enhanced awareness of health hazard prevention, clinical cases of lead poisoning resulting from high‐concentration lead exposure are becoming increasingly rare. Nevertheless, chronic lead exposure is ubiquitous, often occurring through ingestion of industrially contaminated water and food, air pollution, or contact with lead‐containing chemicals (Sun et al. 2023). It typically lacks specific clinical symptoms and is difficult to detect. To clarify the effects of long‐term, low‐concentration lead exposure on the auditory system, we analyzed comprehensive data from 3129 participants in the NHANES database. We found that blood lead concentrations were significantly higher in the hearing loss population compared to the normal‐hearing population, suggesting that chronic lead exposure may be a risk factor for hearing loss. Furthermore, we established a chronic lead exposure mouse model, which directly demonstrated that long‐term lead exposure leads to lead accumulation in the cochlea and irreversible damage to auditory function. However, there are still some limitations. We must acknowledge that our model does not fully replicate chronic low‐level lead exposure in humans, as interspecies differences exist between mice and humans. Currently, no studies have established a direct correlation between blood lead concentrations in humans and mice. Our experimental data can only reflect the impact of lead exposure at a specific dosage on the auditory system of C57BL/6J mice. Future studies will require larger scale and well‐designed prospective cohort research to further clarify the relationship between lead exposure and ARHL.

Aging is a natural and complex physiological process. ARHL is an auditory dysfunction resulting from degenerative changes in the auditory system associated with aging. It is characterized by progressive, irreversible, bilateral, and symmetrical sensorineural hearing loss (Guerrieri et al. 2023). The onset and progression of ARHL are influenced by a variety of factors, including ototoxic drugs, noise exposure, dietary factors, genetic factors, and chronic diseases (Xie et al. 2024; Zeng et al. 2024; Yuan et al. 2024). The pathological changes of ARHL include degeneration of the stria vascularis, spiral ligament, hair cells, afferent nerve fibers, spiral ganglion, and central auditory pathways (Kong et al. 2025). Among these, the degeneration of cochlear hair cells and spiral ganglion cells is considered a primary pathological phenotype (Tawfik et al. 2020; Fischer et al. 2020). In this study, we systematically evaluated pathological changes in the cochlea following chronic low‐level lead exposure. The results indicate that lead accumulation in the cochlea leads to degeneration of cochlear hair cells, synapses, and the spiral ganglion. The free radical theory of aging proposes that aging is a process of oxidative damage caused by the cumulative effects of reactive oxygen species (ROS) (Zhang et al. 2022). When intracellular ROS accumulation surpasses the capacity of endogenous antioxidant mechanisms, it can lead to abnormal mitochondrial metabolism, resulting in impaired oxidative phosphorylation and activation of the apoptotic cascade, ultimately causing cell death (Someya et al. 2008). Mitochondrial dysfunction is not only an early event in ARHL but also one of the causes of its progression, and maintaining normal mitochondrial function may delay the progression of ARHL (Zhang et al. 2024). Previous studies indicate that lead can act directly on mitochondria, causing mitochondrial DNA mutations, accumulation of mitochondrial ROS, and impaired ATP synthesis (Shvachiy, Geraldes, and Outeiro 2023). Through transcriptomic analysis of cochlear tissue from lead‐exposed mice, we confirmed significant alterations in the expression levels of a series of genes related to mitochondrial function. We particularly noted a marked downregulation of the mitochondrial protease LONP1. Furthermore, immunofluorescence staining confirmed significantly reduced LONP1 protein expression levels in the stria vascularis, sensory hair cells, and spiral ganglion. LONP1 dysfunction directly affects mitochondrial protein degradation, and the accumulation of abnormal mitochondrial proteins can lead to cellular damage (Tang et al. 2025; Liu et al. 2024). Consistent with this, we observed a significant increase in the expression of HMGCS2, a substrate of LONP1, in the cochlea. Recent studies have shown that high expression of HMGCS2 induces mitochondrial ROS accumulation and loss of mitochondrial membrane potential, subsequently triggering cellular damage (Wang et al. 2020). Additionally, LONP1 binds to mitochondrial DNA promoter regions, participating in mtDNA replication and transcriptional regulation, while also maintaining genomic stability by degrading aberrantly bound proteins (Xu, Fu, et al. 2022). In summary, our findings suggest that lead exposure may impair LONP1 function, leading to the accumulation of damaged mitochondrial proteins and proteostatic stress in the cochlea, which in turn contributes to cellular degeneration and irreversible auditory dysfunction.

Heavy metal ion exposure has emerged as a significant global public health issue. Reducing heavy metal ion exposure is a foundational, preventive, and strategic task in the field of public health, with direct implications for the life security, health outcomes, and quality of life of billions of people. Lead exposure, characterized by its bioaccumulative nature and severe health hazards, poses a particular threat to vulnerable populations such as children and the elderly. ARHL has emerged as a significant public health challenge in aging societies worldwide. As the population ages, the incidence of ARHL is rising annually. According to the World Health Organization, approximately one‐third of individuals aged 65 and older are affected by hearing loss (Rodríguez‐Valiente et al. 2020; Zimatore et al. 2020). Hearing impairment severely impacts patients' verbal communication and mental health. Although hearing aids and cochlear implants can assist hearing, they present issues such as high costs and variable adaptability (Xing et al. 2021; Tsai Do et al. 2024). For the early intervention of ARHL, understanding its etiology and pathogenesis is crucial. This represents a comprehensive challenge involving multiple fields including public health, neuroscience, and bioengineering. In this study, we have identified chronic low‐level lead exposure as a major risk factor for ARHL, demonstrating that lead accumulation in the cochlea leads to irreversible damage to cochlear tissue cells. Consequently, mitigating the risks of lead exposure requires adopting an interdisciplinary approach, integrating expertise from environmental science, toxicology, and public health. Additionally, implementing stringent regulatory measures, alongside initiatives based on continuous monitoring and early warning systems, is essential. It is particularly critical to strengthen occupational health protections for populations with high exposure risks, such as workers in mining, smelting, battery manufacturing, and e‐waste recycling industries. This article emphasizes the need for a comprehensive and multidisciplinary approach to establish a monitoring system for heavy metal exposure and its health effects.

## 5 Conclusion

Through the integration of large‐scale population data analysis and animal model validation, we demonstrate that chronic low‐concentration lead exposure accelerates the development of ARHL in C57BL/6J mice. Lead exposure mediates damage to cochlear sensory cells via affecting mitochondrial function, leading to irreversible hearing loss. These preclinical findings deepen our understanding of lead exposure‐induced hearing impairment and may stimulate further epidemiological research. They hold profound implications for preventing hearing damage in populations at risk of lead exposure.

## Author Contributions

**Xue Bai:** conceptualization, writing – original draft, visualization, validation, investigation, methodology. **Li‐Hua Cheng:** visualization, validation, investigation, methodology. **Zhi‐Bin Zhou:** investigation, methodology. **Kai‐Lang Zhou:** validation, investigation. **Yan‐Peng Fu:** software, methodology. **Bing Liao:** methodology, formal analysis. **Mei‐Qun Wang:** validation, methodology. **Xu‐Bo Chen:** formal analysis, data curation. **Hong‐Ping Chen:** methodology, data curation. **Yue‐Hui Liu:** writing – review and editing, investigation, funding acquisition. **Kai Xu:** writing – review and editing, project administration, funding acquisition, investigation, conceptualization. All authors read and approved the final manuscript.

## Conflicts of Interest

The authors declare no conflicts of interest.

## Supporting information


**Figure S1:** Lead exposure leads to mitochondrial dysfunction in HEI‐OC1 cells. (A) The ultrastructural morphology of HEI‐OC1 cell in the control group or lead exposure group. (B) Representative fluorescence image of HEI‐OC1 cell stained with JC‐1. (C) Quantitative analysis of the fluorescence intensity of JC‐1 in different treatment groups. ***p < 0.001. Scale in panel A represents for 1 μm. Scale in panel B represents for 20 μm.

## Data Availability

All data that support the findings in this study is available from the corresponding author upon reasonable request.
