# Supplementary material for: Chronic Low‐Level Lead Exposure Causes Auditory Impairment and Accelerates the Progression of Age‐Related Hearing Loss in C57BL/6J Mice
Source: Aging Cell. 2025 Nov 10;25(1):e70297. doi: 10.1111/acel.70297 (PMC12740084; doi:10.1111/acel.70297)
Supplement: Supplementary file 1 — Figure S1: Lead exposure leads to mitochondrial dysfunction in HEI‐OC1 cells. (A) The ultrastructural morphology of HEI‐OC1 cell in the control group or lead exposure group. (B) Representative fluorescence image of HEI‐OC1 cell stained with JC‐1. (C) Quantitative analysis of the fluorescence intensity of JC‐1 in different treatment groups. ***p < 0.001. Scale in panel A represents for 1 μm. Scale in panel B represents for 20 μm. [file ACEL-25-e70297-s001.docx]

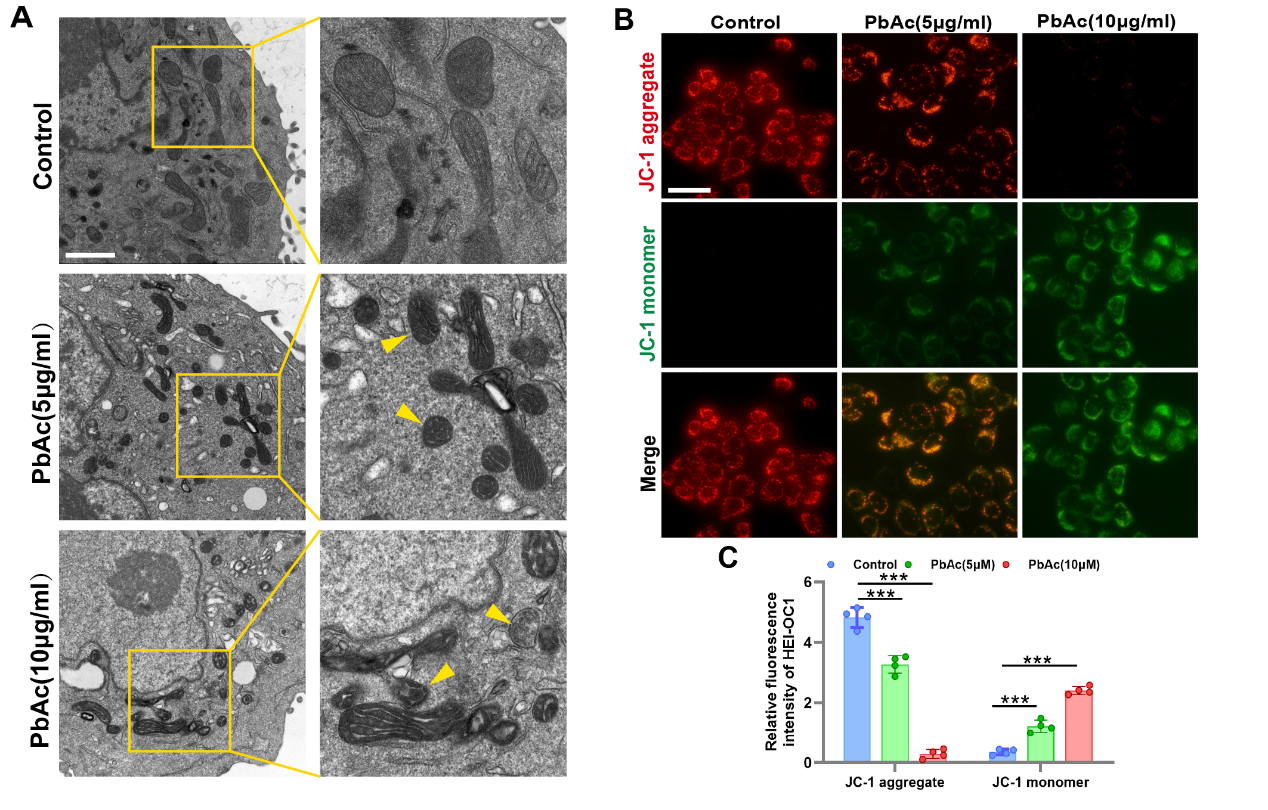


**Fig.S1. Lead exposure leads to mitochondrial dysfunction in HEI-OC1 cells.** (A) The ultrastructural morphology of HEI-OC1 cell in the control group or lead exposure group. (B) Representative fluorescence image of HEI-OC1 cell stained with JC-1. (C) Quantitative analysis of the fluorescence intensity of JC-1 in different treatment groups. *** *P* < 0.001. Scale in panel A represents for 1 μm. Scale in panel B represents for 20 μm.
